# Can a Healthcare Quality Improvement Initiative Reduce Disparity in the Treatment Delay among ST-Segment Elevation Myocardial Infarction Patients with Different Arrival Modes? Evidence from 33 General Hospitals and Their Anticipated Impact on Healthcare during Disasters and Public Health Emergencies

**DOI:** 10.3390/healthcare9111462

**Published:** 2021-10-28

**Authors:** Na Li, Junxiong Ma, Shuduo Zhou, Xuejie Dong, Mailikezhati Maimaitiming, Yinzi Jin, Zhijie Zheng

**Affiliations:** 1 Department of Global Health, School of Public Health, Peking University, Beijing 100871, China; lina2021@bjmu.edu.cn (N.L.); junxiongma@bjmu.edu.cn (J.M.); zhoushuduo@pku.edu.cn (S.Z.); dongxj@bjmu.edu.cn (X.D.); zhengzj@bjmu.edu.cn (Z.Z.); 2 Institute for Global Health and Development, Peking University, Beijing 100871, China; 3 School of Health Humanities, Peking University, Beijing 100871, China; maili@bjmu.edu.cn

**Keywords:** chest pain center accreditation, healthcare quality improvement, door-to-balloon time, arrival mode, ST-segment elevation myocardial infarction

## Abstract

(1) Background: Chest pain center accreditation has been associated with improved timelines of primary percutaneous coronary intervention (PCI) for ST-segment elevated myocardial infarction (STEMI). However, evidence from low- and middle-income regions was insufficient, and whether the sensitivity to improvements differs between walk-in and emergency medical service (EMS)-transported patients remained unclear. In this study, we aimed to examine the association of chest pain center accreditation status with door-to-balloon (D2B) time and the potential modification effect of arrival mode. (2) Methods: The associations were examined using generalized linear mixed models, and the effect modification of arrival mode was examined by incorporating an interaction term in the models. (3) Results: In 4186 STEMI patients, during and after accreditation were respectively associated with 65% (95% CI: 54%, 73%) and 71% (95% CI: 61%, 79%) reduced risk of D2B time being more than 90 min (using before accreditation as the reference). Decreases of 27.88 (95% CI: 19.57, 36.22) minutes and 26.55 (95% CI: 17.45, 35.70) minutes in D2B were also observed for the during and after accreditation groups, respectively. The impact of accreditation on timeline improvement was greater for EMS-transported patients than for walk-in patients. (4) Conclusions: EMS-transported patients were more sensitive to the shortened in-hospital delay associated with the initiative, which could exacerbate the existing disparity among patients with different arrival modes.

## 1. Introduction

ST-segment elevation myocardial infarction (STEMI) is the deadliest and most time-sensitive acute cardiac event. Primary percutaneous coronary intervention (PCI) is the typically recommended treatment for STEMI cases. The door-to-balloon time, also referred to as the in-hospital delay, which denotes the interval from the patient’s arrival at the emergency department to the first inflation of an angioplasty balloon in the occluded coronary artery, is widely used to assess the timeliness of primary PCI [1]. A door-to-balloon time of 90 min or less is given as the Class I (highest level) recommendation according to the American College of Cardiology/American Heart Association (ACC/AHA) and European Society of Cardiology (ESC) guidelines [2,3]. Despite the widespread promulgation and endorsement of the guideline, their translation into clinical practice remains suboptimal. In China, only 32.6% of STEMI patients receive primary PCI within 90 min of arrival [4]. Moreover, there is a very pronounced gap in the door-to-balloon time between walk-in and emergency medical service (EMS)-transported STEMI patients undergoing primary PCI [5,6,7,8]. Therefore, implementation of a healthcare quality improvement initiative, ensuring that hospitals provide timely guideline-recommended clinical practice, is warranted to reduce the in-hospital delay.

A growing strand of studies indicate that accreditation of chest pain centers can facilitate the implementation of strategies to improve healthcare quality for STEMI. Accredited chest pain centers should follow the criteria according to the recommended guideline, accompanied with a number of quality improvement activities (e.g., establishing a standardized monitor system of healthcare performance, carrying out healthcare performance review and feedback). For instance, the United States and Germany have witnessed improvements in the management and clinical outcomes of STEMI patients after an extensive adoption of nationwide programs for chest pain center professional society accreditation [9,10,11,12]. Positive empirical evidence in developed countries has shown that chest pain center accreditation is associated with shortened in-hospital delay for STEMI care [10,13]. However, two key questions remained unanswered: First, does the positive effect of chest pain center accreditation on the in-hospital delay found in other settings apply to China, where the proportion of cases receiving guideline-recommended treatments remains low [4]? Second, given the large disparity in the in-hospital delay among patients with different arrival modes [5,6,8,14,15], is the association between chest pain center accreditation and the in-hospital delay different between walk-in and EMS-transported cases? If so, is chest pain center accreditation widening or narrowing the existing disparities in in-hospital delay? Furthermore, integration of prehospital and hospital care is one of the dimensions of chest pain center accreditation criteria in China. It requires that hospitals should establish a regional collaborative healthcare delivery system that integrates prehospital emergency systems and in-hospital green passages, coordination and division of labor between different hospital departments, and connections between hospitals and community healthcare centers. This also plays a key role in optimizing the capacity of public health infrastructure and/or systems to respond at times of public health emergencies and disasters. Therefore, if chest pain center accreditation is beneficial to timeliness for STEMI patients and other acute cardiac events, it would also contribute to the promptness of triage, transfer, and treatment at times of public health emergencies and disasters.

## 2. Materials and Methods

### 2.1. Study Design and Population

For this study, we utilized data from all the accredited hospitals with PCI capabilities in Beijing during January 2016 to June 2019. For our study, we recruited STEMI patients who met the following criteria:

(1) A discharged diagnosis of STEMI, according to ischemic symptoms, ECG, or positive cardiac markers;
(2) Underwent primary PCI;
(3) Arrived at hospital by either of arrival modes: directly by self or transported via EMS.

Patients were excluded if they had an unknown mode of arrival (*N* = 15), missing hospital arrival time, or implausible door-to-balloon time, such as a negative value or time exceeding 24 h (*N* = 37).

### 2.2. Measurement

#### 2.2.1. Accreditation Status

Hospitals’ accreditation status was authenticated by the National Health Commission of China in April 2018. Chest pain center accreditation is made available to all hospitals in Beijing, and hospitals voluntarily continue to apply for the accreditation in a staggered manner. To June 2019, there were a total of 33 hospitals with accredited chest pain centers in Beijing. Thus, not every hospital had enrolled chest pain patients for 18 months. It takes months to receive accreditation, which is based on a review of information from multiple sources, including self-assessment statements, data reports, and field surveys, jointly led by the National Health Commission of China and a specialist team. Data on individual patients were extracted from the electronic medical systems of each hospital, regarding the data elements which were selected based on the ACC/AHA clinical practice guideline [2]. The accreditation statuses of hospital were grouped as ‘before accreditation’ (had not applied for accreditation), ‘during accreditation’ (were applying for accreditation), and ‘after accreditation’ (had been accredited). Patients who were admitted to hospitals before, during, and after the corresponding date of accreditation were, respectively, classified into the ‘before accreditation’ group, the ‘during accreditation’ group, and the ‘after accreditation’ group.

#### 2.2.2. Outcomes

The primary outcome was the in-hospital delay, measured by the door-to-balloon time and the percentage of cases with door-to-balloon time of more than 90 min. Door-to-balloon time was defined as the interval from the STEMI patient’s arrival at the hospital to inflation of the balloon to restore flow.

#### 2.2.3. Covariates

The EMS-transported patients were defined as patients transported to the hospital by EMS services. Walk-in patients were defined as those arriving at the hospital by self- or private transportation, taxi, public transportation, or walking to the hospital.

Patient-level covariates included age, sex (male or female), and signs and symptoms at presentation: whether the patient had sustainable chest pain (refers to the onset of chest pain that lasts more than 30 min and cannot be relieved by rest), intermittent chest pain (refers to chest pain that lasts a few minutes at a time and can be relieved by rest or elimination of the triggers), chest pain relief, abdominal pain, dyspnea, cardiogenic shock, heart failure, malignant arrhythmia, receiving prehospital cardiopulmonary resuscitation or not, heart rate (beats/min), blood pressure (mmHg), and Killip class (I to IV) [16]. Hospital-level characteristics included time of day of arrival (8:00 a.m. to 16:59 p.m., 17:00 p.m. to 11:59 p.m., 12:00 a.m. to 7:59 a.m.), weekday or off-day arrival (off-days include weekends and Chinese official holidays), hospital level, and region of hospital (urban or suburb). Hospital levels in China are divided into several levels according to the scale, facilities, and ability of hospitals: grade III A, B, and C; grade II A, B, and C; and grade I, with grade IIIA being the highest level. Hospitals of grade IIIA have high-level capacity for primary PCI, and the number of PCIs meets certain requirements, ensuring that emergency PCI operations are performed 24 h a day.

### 2.3. Data Analysis

The characteristics of patients and hospitals and the in-hospital delay were compared between walk-in and EMS-transported patients, with the use of the chi-square test for categorical variables and the *Kruskal–Wallis* test for continuous variables. Categorical variables are presented as counts and percentages. Quantitative variables are expressed as means ± standard deviations (SDs) or medians and interquartile ranges. *p* values of less than 0.05 were considered to indicate statistical significance. We describe the median of door-to-balloon times and the percentage of patients for whom door-to-balloon times were 90 min or more across arrival modes and accreditation status.

To account for clustering of patients within hospitals, we employed generalized linear mixed models with a random effect term for the hospital to examine the associations of arrival mode and accreditation status with in-hospital delay (Model 1). The logistic regression was performed for the percentage of cases with door-to-balloon time of more than 90 min, and the effect estimates are reported as odds ratios (ORs) and 95% CI. For the door-to-balloon time, the effect estimates were calculated from the linear regression and reported as changes in minutes. Variables included in the models were selected based on their physiological relevance and potential to be associated with outcomes. We initiated the model development with a crude model (no adjustment) and then added a range of covariates into the regression models based on previous studies in the literature [14,17,18,19]. All the models were adjusted for sex, age, signs and symptoms at presentation, heart rate, blood pressure and Killip class, arrival mode, time of day of arrival, day of arrival, class of hospital, region of hospital, and accreditation status. To examine the modification effect of arrival mode in the association of accreditation status with the in-hospital delay, an interaction term of arrival mode and accreditation status was incorporated into the model (Model 2). All the models were adjusted for covariates, with *p* < 0.05 considered the level of statistical significance. Hospital was added as a random effect term in the models to adjust for unobserved hospital-level factors. All statistical analyses were performed using R software.

## 3. Results

### 3.1. Characteristics of Patients and Hospitals

A total of 4186 STEMI patients undergoing PCI from 33 hospitals were enrolled in this study, including 1284 (30.7%) EMS-transported patients and 2902 (69.3%) walk-in patients. The overall median age of the patients was 60 years, and 80.5% were men. The patients admitted to hospitals before, during, and after accreditation accounted for 43.2%, 13.7, and 43.1%, respectively. A comparison of patient- and hospital-level characteristics by arrival mode is presented in Table 1. In general, compared with walk-in patients, EMS-transported patients had lower heart rate (73.5 versus 75.9 beats/min, *p* < 0.001), lower systolic blood pressure (122.7 versus 132.5 mmHg, *p* < 0.001), lower diastolic blood pressure (76 versus 81.3 mmHg, *p* < 0.001), and lower rate of Killip I status (81.3% versus 89.7%, *p* < 0.001). Regarding hospital-level characteristics, 72.2% of EMS-transported patients arrived at urban hospitals, slightly larger than the proportion of walk-in patients (67.1%, *p* < 0.001).

The in-hospital delay also varied in the two groups of patients (Table 2) and by different status (Figure S1 and Table S1). The median door-to-balloon time (70 versus 85 min, *p* < 0.001) and the percentage of cases with door-to-balloon time more than 90 min (26.7% versus 43.9%, *p* < 0.001) in EMS-transported patients were lower than those in walk-in patients.

### 3.2. Association between Accreditation Status and In-Hospital Delay

**Figure 1:**
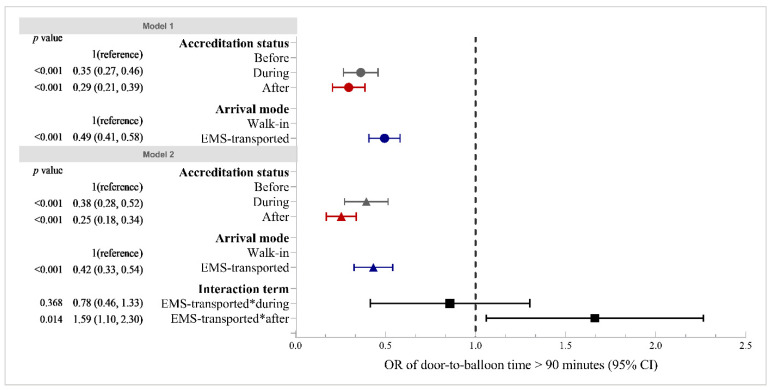
Associations of in-hospital delay (door-to-balloon time of >90 min) with accreditation status and arrival mode. Abbreviations: 95% CI, 95% confidence interval; EMS, emergency medical service. Notes: Both Models 1 and 2 were adjusted for sex, age, signs and symptoms at presentation, heart rate, blood pressure and Killip class, arrival mode, time of day of arrival, day of arrival, class of hospital, region of hospital, and accreditation status. Model 1 did not contain an interaction term. Model 2 included an interaction term of arrival mode and accreditation status. The OR value of the interaction term indicates that the decreased likelihood of door-to-balloon time being more than 90 min associated with accreditation status for EMS-transported patients was (OR − 1) ∗ 100%-more than that for walk-in patients.

Figure 1 shows the results of generalized linear mixed models of the likelihood of door-to-balloon time being more than 90 min. According to the full adjustment model, compared with the ‘before accreditation’ group, the risk of the door-to-balloon time being more than 90 min was significantly lower in both the ‘during accreditation’ group (OR: 0.35, 95% CI: 0.27, 0.46) and the ‘after accreditation’ group (OR: 0.29, 95% CI: 0.21, 0.39). Arrival by EMS was associated with a lower risk of the door-to-balloon time being more than 90 min, compared with arrival by self (OR: 0.49, 95% CI: 0.41, 0.58). The results generated by crude models are presented in Table S2.

**Figure 2:**
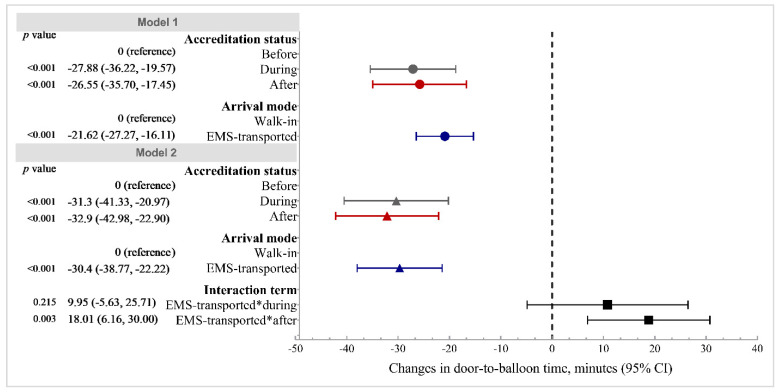
Associations of door-to-balloon time with accreditation status and arrival mode. Abbreviations: 95% CI, 95% confidence interval; EMS, emergency medical service. Notes: Both Model 1 and 2 were adjusted for sex, age, signs and symptoms at presentation, heart rate, blood pressure and Killip class, arrival mode, time of day of arrival, day of arrival, class of hospital, region of hospital, and accreditation status. Model 1 did not contain an interaction term. Model 2 included an interaction term of arrival mode and accreditation status. The β value of the interaction term indicates that the decrease in door-to-balloon time associated with accreditation status for EMS-transported patients was β minutes more than that for walk-in patients.

Figure 2 shows the results of generalized linear mixed models of the door-to-balloon time. Compared with the ‘before accreditation’ group, we observed significant decreases of 27.88 (95% CI: −36.22, −19.57) minutes and 26.55 (95% CI: −35.70, −17.45) minutes for the ‘during accreditation’ group and ‘after accreditation’ group, respectively. Those transported by EMS exhibited a 21.62 (95% CI: −27.27, −16.11) minute decrease in door-to-balloon time compared with walk-in patients. The results generated by crude models are presented in Table S3.

### 3.3. Association between Accreditation Status and Disparity of In-Hospital Delay across Arrival Modes

After adding the interaction term between accreditation status and arrival by EMS, the negative associations between ‘after accreditation’ status and in-hospital delay remained statistically significant and had larger coefficient sizes in the likelihood of door-to-balloon time being more than 90 min (OR: 0.25, 95% CI: 0.18, 0.34) and the door-to-balloon time (β: −32.90, 95% CI: −42.98, −22.90). In terms of differences in arrival mode, the coefficient sizes of arrival by EMS were also larger in the in-hospital delay: the likelihood of door-to-balloon time being more than 90 min (OR: 0.42, 95% CI: 0.33, 0.54) and the door-to-balloon time (β: −30.40, 95% CI: −38.77, −22.22).

The OR of the interaction term of arrival by EMS and ‘during accreditation’ was statistically insignificant, suggesting that arrival by EMS did not modify the effect of accreditation process on in-hospital delay. The OR of the interaction term of arrival by EMS and ‘after accreditation’ was 1.59 (95% CI: 1.10, 2.30), indicating that completed accreditation widened the disparity in the in-hospital delay between walk-in and EMS-transported patients. The impact of completed accreditation on the likelihood of door-to-balloon time being more than 90 min for EMS-transported patients was 59% greater than that for walk-in patients (Figure 1). Similar patterns were also found for the door-to-balloon time. A β value of 18.01 was found for the interaction term of arrival by EMS and ‘after accreditation’, suggesting that the reduction in the door-to-balloon time associated with completed accreditation for EMS-transported patients was 18.01 min more than that for walk-in patients (Figure 2).

## 4. Discussion

To the best of our knowledge, this was the first study to identify the modifying role of arrival mode in associations of chest pain center accreditation with in-hospital delay. Our results reflect an overall significant reduction in the in-hospital delay among STEMI patients after hospitals were accredited; however, the improvement was inconsistent between walk-in and EMS-transported patients. Our findings suggest that the healthcare quality improvement initiative may widen the disparity in treatment delay for patients with different arrival modes, providing implications for the optimization of implementation strategies for the continuous quality improvement of healthcare for acute chest pain.

Our results showed that compared with ‘before accreditation’, both ‘during accreditation’ and ‘after accreditation’ statuses were associated with lower prevalence of in-hospital delay among STEMI patients undergoing primary PCI. This finding is generally consistent with studies conducted in other regions, which indicated that chest pain center accreditation was associated with improved processes and outcomes for patients with STEMI [9,12,20]. Generally, chest pain center accreditation is a hospital-based, multifaceted, continuous quality improvement initiative from a multidisciplinary approach; it can be an efficient way to improve the in-hospital process and is of great significance to shortening the treatment time for STEMI patients. Furthermore, the negative associations of completed accreditation (‘after accreditation’ status) with in-hospital delay were more pronounced among EMS-transported patients than among walk-in patients.

There are several potential explanations for the observed disparity in sensitivity to chest pain center accreditation efforts. First, establishment of a regional collaborative healthcare network from a multiagency approach was emphasized in the current practice for achieving chest pain center accreditation criteria. Delivery of EMS always occurs across multiple sectors, including emergency departments, centers for prehospital care, ambulance stations, and day care or primary healthcare centers, and it requires at least two different services, with each service provided by different settings. Care coordination is critical for the delivery of EMS to ensure that healthcare professionals interact with each other to provide timely and efficient healthcare. A large number of studies have also shown that the transmission of prehospital electrocardiogram and prehospital diagnosis is the primary basis for a hospital to decide whether to bypass the emergency department and cardiac care unit, which can reduce the in-hospital delay for EMS-transported STEMI patients [21,22,23]. Pre-hospital ECGs going directly to the hospital by bypassing the emergency department and even coronary care unit is a general practice for achieving this objective. The EMS-transported patients also had greater and significantly faster receipt of initial reperfusion therapies [24]. Second, the condition of patients who are transported to hospital by ambulance is generally considered to be more urgent and more severe. They are given more attention and a higher medical priority when arriving at emergency departments. As soon as the ambulance arrives, prompt diagnosis, triage, and treatment are provided by healthcare professionals who are on stand-by in advance. Third, for walk-in patients, they have to undergo the normal medical procedures after arriving at the hospital, such as consulting, registering, paying, and even waiting for treatment. They cannot get the rapid and priority healthcare that patients transported by EMS can have. As a result, the time interval between their arrival at the gate of the hospital and initiation of reperfusion is extended.

In addition to the integration of prehospital and hospital care, implementation measurements required by the chest pain center accreditation criteria could also provide a plausible explanation for the mitigation of in-hospital delay among STEMI patients requiring primary PCI. Accredited hospitals continuously report data on individual patients for quality monitoring and assessment. The indicators for measuring clinical performance quarterly and annually are reported, and a ranking is calculated based on the percentile of each indicator and a weighted composite score. In terms of auditing and feedback regarding clinical performance, an improvement in adherence to the guideline recommendations is facilitated through monthly and quarterly hospital-specific performance feedback reports. The hospital-specific data are compared against a variety of internal and external benchmarks, including the temporal trend in performance and comparison points to regional or national performance thresholds, led by the National Health Commission of China. A series of regular meetings and case management and case study meetings are carried out at least once every quarter to share ‘best practice’ clinical support tools in hospitals. Regarding educational outreach to clinicians, routine educational programs are organized, and the contents of training include rules and guidelines for chest pain center construction, clinical skills for the diagnosis and treatment of STEMI cases, and standardization and guidelines for real-time data reporting. These dimensions of implementation measurements required by the chest pain center accreditation criteria could also benefit the development of public health capacity and capability to respond to public health emergencies by saving resources for triage, promoting efficiency of transfer, and optimizing timeliness of treatment.

The current findings suggest that some attention should be channeled to walk-in patients in order to eliminate the inequality of the implementation effect of the healthcare quality improvement between patients with different arrival modes. The strategies to deal with this disparity might include, but are not restricted to, the following suggestions. From the patient level, health education on recognition of the onset symptoms of STEMI and awareness of seeking treatment by calling EMS should be encouraged and perhaps conducted by community healthcare centers and hospital-based chest pain centers. The existing evidence indicates that wider use of EMS by patients with acute chest pain may offer a considerable opportunity for improvement in public health [14,21,22,25,26]. From the level of healthcare professionals, physicians, general practitioners, and nurses in emergency department should pay close attention to walk-in patients whose main complaint is chest pain. On the one hand, healthcare professionals should improve capacity for rapid diagnostics and triage of STEMI requiring primary PCI. On the other hand, the hospital could set up a green channel to optimize the ambulatory treatment process for them so as to buy time for healthcare professionals. From the hospital level, it is warranted to reinforce the information sharing and communication between the emergency and cardiology departments and establish a multidisciplinary coordinated team of healthcare professionals for comprehensive triage, treatment, and transfer of STEMI cases.

There were some limitations to this study. First, the nature of the cross-sectional design of this study restricted us to making causal inferences between the chest pain center accreditation and decreased in-hospital delay. Rather, the associations found in the present study underscore the need for research to capitalize on chest pain center accreditation to mitigate in-hospital delay. Second, this study included patients who were undergoing PCI; therefore, the results cannot be generalized to all patients with STEMI. Third, we were unable to adjust for medical history and socioeconomic indicators (e.g., income, educational attainment, marriage status, etc.) due to the unavailability of relevant data for the patients. However, a previous publication suggested that these variables are not associated with in-hospital delay but might be associated with pre-hospital delay and mortality [27]. Moreover, in terms of measuring the effect on the in-hospital delay rather than clinical outcomes, we adjusted for Killip classification, which is positively associated with medical history of patients [16,28] and could account for the partial confounding effect of medical history. Finally, although our analysis included all 33 centers accredited during January 2016 to June 2019 in Beijing, it is inevitable to introduce heterogeneity regarding the quality of data collection. Voluntary participation of hospitals made it difficult for us to compare with those not seeking accreditation.

## 5. Conclusions

Among STEMI patients undergoing primary PCI, EMS-transported patients were more sensitive to the shortened in-hospital delay associated with chest pain center accreditation efforts. This effect might exacerbate the existing disparity in in-hospital delay among patients with different arrival modes. Thus, more attention should be paid to walk-in patients and more strategies for increasing the utilization of EMS should be considered in further healthcare quality improvement.

## Figures and Tables

**Table 1:** Patient- and hospital-level characteristics of participants.

Characteristics	Overall	Arrival Mode		*p* Value
		EMS-Transported	Walk-In	
Number of admissions, *n* (%)	4186 (100)	1284 (30.7)	2902 (69.3)	
Patient-level				
Sex, *n* (%)				
Male	3368 (80.5)	1025 (79.8)	2343 (80.7)	0.521
Female	818 (19.5)	259 (20.2)	559 (19.3)	
Age, median (q1, q3)	60 (52, 69)	61 (53, 70)	60 (51, 69)	0.010
Sustainable chest pain, *n* (%)	2385 (57.0)	858 (66.8)	1527 (52.6)	<0.001
Intermittent chest pain, *n* (%)	545 (13.0)	136 (10.6)	409 (14.1)	0.001
Chest pain relief, *n* (%)	27 (0.6)	10 (0.8)	17 (0.6)	0.008
Abdominal pain, *n* (%)	34 (0.8)	6 (0.5)	28 (1.0)	0.182
Dyspnea, *n* (%)	38 (0.9)	9 (0.7)	29 (1.0)	0.644
Shock, *n* (%)	31 (0.7)	21 (1.6)	10 (0.3)	<0.001
Heart failure, *n* (%)	15 (0.4)	10 (0.8)	5 (0.2)	0.007
Malignant arrhythmia, *n* (%)	33 (0.8)	22 (1.7)	11 (0.4)	<0.001
CPR, *n* (%)	17 (0.4)	13 (1.0)	4 (0.1)	<0.001
Heart rate (beats/min), mean (SD)	75.1 (18.0)	73.5 (19.2)	75.9 (17.3)	<0.001
Systolic blood pressure (mm Hg), mean (SD)	129.5 (27.7)	122.7 (27.5)	132.5 (27.3)	<0.001
Diastolic blood pressure (mm Hg), mean (SD)	79.6 (18.0)	76.0 (18.4)	81.3 (17.6)	<0.001
Killip class, *n* (%)				
I	3324 (87.1)	974 (81.3)	2350 (89.7)	<0.001
II	334 (8.8)	136 (11.4)	198 (7.6)	
III	39 (1.0)	24 (2.0)	15 (0.6)	
IV	120 (3.1)	64 (5.3)	56 (2.1)	
Hospital-level				
Region, *n* (%)				
Urban	2875 (68.7)	927 (72.2)	1948 (67.1)	0.001
Suburb	1311 (31.3)	357 (27.8)	954 (32.9)	
Hospital level, *n* (%)				
Grade III A	3262 (77.9)	985 (76.7)	2277 (78.5)	0.223
Non-grade III A	924 (22.1)	299 (23.3)	625 (21.5)	
Day of arrival, *n* (%)				
Weekday	2788 (66.6)	856 (66.7)	1932 (66.6)	0.982
Off-day	1398 (33.4)	428 (33.3)	970 (33.4)	
Time of day of arrival, *n* (%)				
8 a.m. to 16:59 p.m.	1861 (45.6)	588 (46.1)	1273 (45.3)	0.726
17 p.m. to 11:59 p.m.	1113 (27.3)	351 (27.5)	762 (27.1)	
12 a.m. to 7:59 a.m.	1110 (27.2)	336 (26.4)	774 (27.6)	
Accreditation status, *n* (%)				
Before	1809 (43.2)	576 (44.9)	1233 (42.5)	<0.001
During	574 (13.7)	217 (16.9)	357 (12.3)	
After	1803 (43.1)	491 (38.2)	1312 (45.2)	

Abbreviations: EMS, emergency medical service; CPR, cardiopulmonary resuscitation; q1, the first quartile; q3, the third quartile; SD, standard deviation. Notes: Sustainable chest pain refers to the onset of chest pain that lasts more than 30 min and cannot be relieved by rest. Intermittent chest pain refers to chest pain that lasts a few minutes at a time and can be relieved by rest or elimination of the triggers.

**Table 2:** Door-to-balloon time in patients with different arrival modes.

Outcome	Overall	Arrival Mode		*p* Value
		EMS-Transported	Walk-In	
Door-to-balloon time (minutes), median (q1, q3)	81 (62, 105)	70 (52, 90)	85 (67, 111)	<0.001
Door-to-balloon time >90 min, *n* (%)				
No	2568 (61.3)	941 (73.3)	1627 (56.1)	<0.001
Yes	1618 (38.7)	343 (26.7)	1275 (43.9)	

Abbreviations: EMS, emergency medical service; q1, the first quartile; q3, the third quartile.

## Data Availability

The data presented in this study are available on request from the corresponding author. The data are not publicly available due to privacy.

## Supplementary Materials

The following are available online at https://www.mdpi.com/article/10.3390/healthcare9111462/s1, Figure S1: Median and interquartile range of door-to-balloon time by accreditation status and arrival mode. Table S1: Median and interquartile range of door-to-balloon time (minutes) by accreditation status and arrival mode. Table S2: Odds ratio (OR) and 95% confidence interval (95% CI) of in-hospital delay associated with accreditation status and transfer mode. Table S3: Changes in door-to-balloon time and 95% confidence interval (95%CI) associated with accreditation status and transfer mode. Table S4: Odds ratio (OR) and 95% confidence interval (95% CI) of in-hospital delay associated with accreditation status by arrival mode. Table S5: Changes in door-to-balloon time and 95% confi-dence interval (95%CI) associated with accreditation status by arrival mode. Table S6: Odds ratio (OR) and 95% confidence interval (95% CI) of covariates obtained from fully adjusted models. Table S7: Changes in door-to-balloon time (minutes) and 95% confidence interval (95%CI) of co-variates obtained from fully adjusted models.
