# Supplementary material for: Can a Healthcare Quality Improvement Initiative Reduce Disparity in the Treatment Delay among ST-Segment Elevation Myocardial Infarction Patients with Different Arrival Modes? Evidence from 33 General Hospitals and Their Anticipated Impact on Healthcare during Disasters and Public Health Emergencies
Source: Healthcare (Basel). 2021 Oct 28;9(11):1462. doi: 10.3390/healthcare9111462 (PMC8621169; doi:10.3390/healthcare9111462)
Supplement: Supplementary file 1 [file healthcare-09-01462-s001.zip › healthcare-1392369-supplementary.pdf]

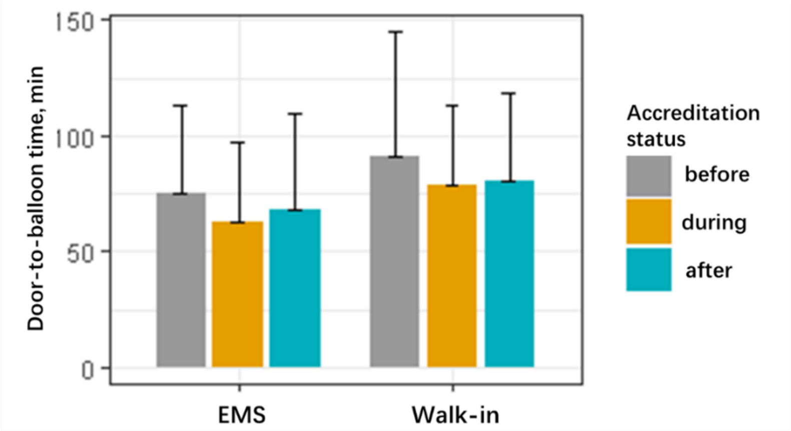

Figure S1. Median and interquartile range of door-to-balloon time by accreditation status and arrival mode.

Table S1. Median and interquartile range of door-to-balloon time (minutes) by accreditation status and arrival mode.

Accreditation status	Arrival mode	
	EMS	Walk-in
Before	75 (38.2)	91 (54)
During	63 (34)	79 (34)
After	68 (41.5)	80 (38)

Table S2. Odds ratio (OR) and 95% confidence interval (95% CI) of in-hospital delay associated with accreditation status and transfer mode.

	Crude model			Adjusted model		
	OR	95% CI	*p* value	OR	95% CI	*p* value
**Accreditation status**						
before	Reference (OR=1)			Reference (OR=1)		
during	0.36	0.28, 0.46	<0.001	0.35	0.27, 0.46	<0.001
after	0.31	0.24, 0.41	<0.001	0.29	0.21, 0.39	<0.001
**Arrival mode**						
walk-in	Reference (OR=1)			Reference (OR=1)		
EMS	0.52	0.45, 0.61	<0.001	0.49	0.41, 0.58	<0.001

Table S3. Changes in door-to-balloon time and 95% confidence interval (95%CI) associated with accreditation status and transfer mode.

	Crude model			Adjusted model		
	β	95%CI	*p value*	β	95%CI	*p value*
**Accreditation status**						
before	Reference (β=0)			Reference (β=0)		
during	-27.48	-35.23, -19.74	<0.001	-27.87	-36.22, -19.57	<0.001
after	-27.56	-35.72, -19.37	<0.001	-26.55	-35.70, -17.45	<0.001
**Arrival mode**						
walk-in	Reference (β=0)			Reference (β=0)		
EMS	-21.79	-26.89, -16.72	<0.001	-21.62	-27.27, -16.10	<0.001

Table S4. Odds ratio (OR) and 95% confidence interval (95% CI) of in-hospital delay associated with accreditation status by arrival mode.

	OR	95% CI	*p value*
Walk-in-before	reference		
Walk-in-during	0.38	0.28, 0.52	<0.001
Walk-in-after	0.25	0.18, 0.34	<0.001
EMS-before	0.42	0.33, 0.54	<0.001
EMS-during	0.33	0.21, 0.53	0.368
EMS-after	0.67	0.51, 0.89	0.014

Table S5. Changes in door-to-balloon time and 95% confidence interval (95%CI) associated with accreditation status by arrival mode.

	β	95% CI	*p value*
Walk-in-before	reference		
Walk-in-during	-31.10	-41.33, -20.97	<0.001
Walk-in-after	-32.90	-42.98, -22.90	<0.001
EMS-before	-30.40	-38.77, -22.22	<0.001
EMS-during	-20.44	-33.95, -6.93	0.215
EMS-after	-12.39	-29.18, -11.71	0.003

Table S6. Odds ratio (OR) and 95% confidence interval (95% CI) of covariates obtained from fully adjusted models.

Variables	OR	95%CI	
		Lower limit	Upper limit
**Hospital level**			
Grade III A	1 (Reference)		
Non-grade III A	1.265	0.576	2.776
**Time of day of arrival**			
8 a.m. to 16:59 p.m.	1 (Reference)		
17 p.m. to 11:59 p.m.	1.324	1.105	1.586
12 a.m. to 7:59 a.m.	1.374	1.148	1.644

**Day of arrival**			
Weekday	1 (Reference)		
Off-day	1.282	1.095	1.500
**Region**			
Urban	1 (Reference)		
Suburb	0.766	0.330	1.779
**Sex**			
Male	1 (Reference)		
Female	1.157	0.951	1.408
**Age**	1.006	1.000	1.013
**Sustainable chest pain**			
No	1 (Reference)		
Yes	0.408	0.267	0.623
**Intermittent chest pain**			
No	1 (Reference)		
Yes	0.526	0.332	0.835
**Chest pain relief**			
No	1 (Reference)		
Yes	1.596	0.604	4.216
**Abdominal pain**			
No	1 (Reference)		
Yes	0.533	0.219	1.295
**Dyspnea**			
No	1 (Reference)		
Yes	0.889	0.407	1.942
**Shock**			
No	1 (Reference)		
Yes	0.942	0.352	2.522
**Heart failure**			
No	1 (Reference)		
Yes	1.488	0.452	4.894
**Malignant arrhythmia**			
No	1 (Reference)		
Yes	0.723	0.284	1.840
**Systolic blood pressure**	1.005	1.001	1.010
**Diastolic blood pressure**	0.998	0.991	1.005
**Killip class**			
I	1 (Reference)		
II	0.984	0.732	1.321
III	1.571	0.720	3.429
IV	1.609	0.997	2.595

Table S7. Changes in door-to-balloon time (minutes) and 95% confidence interval (95%CI) of covariates obtained from fully adjusted models.

Variables	β	95%CI	
		Lower limit	Upper limit
**Hospital level**			
Grade III A	1 (Reference)		
Non-grade III A	9.63	-12.56	32.06
**Time of day of arrival**			
8 a.m. to 16:59 p.m.	1 (Reference)		
17 p.m. to 11:59 p.m.	-1.75	-7.71	4.16
12 a.m. to 7:59 a.m.	3.67	-2.28	9.57
**Day of arrival**			
Weekday	1 (Reference)		
Off-day	2.14	-3.07	7.31
**Region**			
Urban	1 (Reference)		
Suburb	-10.86	-34.82	12.79
**Sex**			
Male	1 (Reference)		
Female	3.94	-2.58	10.48
**Age**	0.11	-0.11	0.32
**Sustainable chest pain**			
No	1 (Reference)		
Yes	-14.06	-27.73	-0.65
**Intermittent chest pain**			
No	1 (Reference)		
Yes	1.60	-13.15	16.23
**Chest pain relief**			
No	1 (Reference)		
Yes	97.49	65.17	129.26
**Abdominal pain**			
No	1 (Reference)		
Yes	-9.45	-39.24	20.00
**Dyspnea**			
No	1 (Reference)		
Yes	21.37	-4.95	47.84
**Shock**			
No	1 (Reference)		
Yes	31.70	0.81	62.54
**Heart failure**			
No	1 (Reference)		
Yes	-5.89	-45.74	34.03
**Malignant arrhythmia**			
No	1 (Reference)		

Yes	-14.97	-42.40	12.65
**Systolic blood pressure**	0.12	-0.03	0.27
**Diastolic blood pressure**	-0.08	-0.31	0.15
**Killip class**			
I	1 (Reference)		
II	3.15	-6.02	12.42
III	8.30	-15.92	32.37
IV	6.33	-8.92	21.63
